# Binary classification of users of electronic cigarettes and smokeless tobacco through biomarkers to assess similarity with current and former smokers: machine learning applied to the population assessment of tobacco and health study

**DOI:** 10.1186/s12889-023-15511-3

**Published:** 2023-03-29

**Authors:** Hiromi Ohara, Shigeaki Ito, Yuichiro Takanami

**Affiliations:** Scientific Product Assessment Center, Japan Tobacco Inc, 6-2 Umegaoka, Aoba-ku, Yokohama, 227-8512 Kanagawa Japan

**Keywords:** Population study, Smoking, E-cigarette, Oral tobacco, Supervised learning, Biomarker of exposure, Biomarker of potential harm.

## Abstract

**Background:**

Exposure to harmful and potentially harmful constituents in cigarette smoke is a risk factor for cardiovascular and respiratory diseases. Tobacco products that could reduce exposure to these constituents have been developed. However, the long-term effects of their use on health remain unclear. The Population Assessment of Tobacco and Health (PATH) study is a population-based study examining the health effects of smoking and cigarette smoking habits in the U.S. population. Participants include users of tobacco products, including electronic cigarettes and smokeless tobacco. In this study, we attempted to evaluate the population-wide effects of these products, using machine learning techniques and data from the PATH study.

**Methods:**

Biomarkers of exposure (BoE) and potential harm (BoPH) in cigarette smokers and former smokers in wave 1 of PATH were used to create binary classification machine-learning models that classified participants as either current (BoE: N = 102, BoPH: N = 428) or former smokers (BoE: N = 102, BoPH: N = 428). Data on the BoE and BoPH of users of electronic cigarettes (BoE: N = 210, BoPH: N = 258) and smokeless tobacco (BoE: N = 206, BoPH: N = 242) were input into the models to investigate whether these product users were classified as current or former smokers. The disease status of individuals classified as either current or former smokers was investigated.

**Results:**

The classification models for BoE and BoPH both had high model accuracy. More than 60% of participants who used either one of electronic cigarettes or smokeless tobacco were classified as former smokers in the classification model for BoE. Fewer than 15% of current smokers and dual users were classified as former smokers. A similar trend was found in the classification model for BoPH. Compared with those classified as former smokers, a higher percentage of those classified as current smokers had cardiovascular disease (9.9–10.9% vs. 6.3–6.4%) and respiratory diseases (19.4–22.2% vs. 14.2–16.7%).

**Conclusions:**

Users of electronic cigarettes or smokeless tobacco are likely to be similar to former smokers in their biomarkers of exposure and potential harm. This suggests that using these products helps to reduce exposure to the harmful constituents of cigarettes, and they are potentially less harmful than conventional cigarettes.

## Background

Cigarette smoking has been reported to increase the risk of several diseases, including chronic obstructive pulmonary disease (COPD) and cardiovascular disease (CVD) [1]. These diseases have been suggested to be caused by the harmful constituents of cigarette smoke [2, 3]. Tobacco companies have been developing products that may reduce disease risks because they emit fewer harmful and potentially harmful constituents [4]. Cigarette smokers switching to these products showed reductions in their biomarkers of exposure (BoE), which are derived from constituents in tobacco smoke [5–13]. Biomarkers of potential harm (BoPH) (e.g., oxidative stress, inflammation, lipid metabolism, and platelet activation/coagulation) have also been reported to be closer to those of non-smokers or former smokers in those who switch from conventional cigarettes to tobacco heating systems [14, 15], and nonconventional vapor products [16]. However, further studies are needed to verify the risk reduction achieved by switching to these products, including with a sufficiently large sample and good background information.

The Population Assessment of Tobacco and Health (PATH) study is a joint project of the Food and Drug Administration (FDA) and the National Institutes of Health (NIH), and is one of the largest studies that track tobacco product use and health effects over time [17]. In the PATH study, questionnaire data on smoking status and health status, biomarkers of exposure and potential harm, and other background information were obtained from participants and registered with the Inter-University Consortium for Political and Social Research (ICPSR). The PATH study includes dual and exclusive users of potentially lower-risk products (e.g., electronic cigarettes and smokeless tobacco) as well as current and former smokers and non-smokers. The study data will therefore allow for a more extensive analysis of tobacco smoke exposure and the biological effects of using potentially lower-risk products. Several reports have compared biomarkers among smokers and users of electronic cigarettes and smokeless tobacco products using the PATH study data [18–20], making statistical group comparisons for each biomarker. These statistical methods may yield different comparisons and different results for some biomarkers within the same report: for example, the concentrations of nicotine metabolites and tobacco-specific nitrosamines were higher in users of smokeless products than in current smokers, while those of polycyclic aromatic hydrocarbons and volatile organic compounds were lower [18].

Machine learning optimizes technologically advanced computational power and statistical tools for the analysis of big data, with advanced algorithms capable of handling multicollinearity, nonlinearity, and higher-order interactions among variables [21]. In general, supervised learning techniques are used in classification models. The model is trained with labeled data to properly classify non-labeled data. Where binary classification is used, the model shows whether the new observations are closer to one or another option based on the features of the training dataset. Previous studies reported that users of electronic cigarettes and smokeless tobacco could have levels of several biomarkers that are closer to those found in non-smokers or former smokers than current smokers. However, these studies evaluated the biomarkers individually, and not comprehensively. Machine learning can evaluate the difference between participants by integrating all tested biomarkers. This study aimed to develop two classification models to discriminate between current and former smokers based on their biomarkers of exposure and potential harm. The classification models that had learned the features of current and former smokers were then used to evaluate markers from users of electronic cigarettes and smokeless tobacco products to see if they were similar to either current or former smokers. It is also possible to evaluate the differences in disease prevalence in individuals, including users of electronic cigarettes and smokeless tobacco products, between those classified as current and former smokers. This may suggest whether electronic cigarettes and smokeless tobacco products genuinely pose a potentially lower risk to health.

## Methods

### Study design

We used data for adults (18 to 90 years old) from the public-use files (ICPSR (a), ICPSR 36,498) and restricted-use files (ICPSR (b), ICPSR 36,231) from Wave 1 (September 12, 2013 to December 14, 2014) of the PATH Study. To protect the rights, welfare, and well-being of all human participants in this study, the Westat Institutional Review Board approved the study design and protocol, and the Office of Management and Budget approved data collection. The detailed study design and data collection of the PATH study have previously been published [17].

### Participants

Groups were defined using self-reports in the PATH study questionnaire. Participants who reported smoking cigarettes daily but not using any other tobacco products were defined as exclusive cigarette smokers (CS group). Exclusive users of electronic cigarettes and smokeless tobacco products were defined similarly (EPRODS and SMKLS groups). Participants who reported using both conventional and electronic cigarettes, or conventional cigarettes and smokeless tobacco products, were defined as dual users (dual-EPRODS and dual-SMKLS groups). People who reported that they had quit smoking and did not use any tobacco products were defined as former smokers (ExSM group). We also extracted information on age, gender, whether they drank alcohol, lived in urban or rural areas, and health status (cardiovascular disease; respiratory disease; high blood pressure, high cholesterol, and diabetes) from the questionnaire data. For health status, those who answered “Yes” to the question “Has a doctor, nurse or other health professional told you that you have COPD?” were considered to have COPD. People who answered “Yes” to questions on any of COPD, chronic bronchitis, emphysema, or other pulmonary or respiratory diseases were considered to have a respiratory disease. Anyone who answered “Yes” to questions about congestive heart failure, stroke, heart attack, or some other heart condition were considered to have CVD. A total of 2707 participants were included in the analysis for biomarkers of exposure, and 3466 in the analysis for biomarkers of potential harm.

### Biomarkers

For the biomarkers of exposure included in the restricted data collected by the PATH study, we selected those that corresponded to harmful and potentially harmful constituents listed by the FDA [4] and evaluated in previous tobacco evaluation studies [6, 10]. These included 4-(methylnitrosamino)-4-(3-pyridyl)-1-butanol (NNAL), N’-nitrosonornicotine (NNNT), total nicotine equivalents (TNE7), N-acetyl-S-(2-carboxyethyl)-L-cysteine (CEMA), N-acetyl-S-(2-hydroxyethyl)-L-cysteine (HEMA), N-acetyl-S-(3-hydroxypropyl)-L-cysteine (HPMA), N-acetyl-S-(phenyl)-L-cysteine (PMA), 1-naphthol or 1-hydroxynaphthalene (P01), 2-naphthol or 2-hydroxynaphthalene (P02), and 1-hydroxypyrene (P10). For the biomarkers of potential harm, we used 8-isoprostane (8PGFT), high-sensitivity C-reactive protein (hsCRP), interleukin 6 (IL6), soluble intercellular adhesion module (sICAM), and fibrinogen (FIB). The creatinine correction was used for each participant for biomarkers in urine samples to adjust for daily excretion.

### Machine learning

To predict the exposed constituent and biological effects of tobacco smoke from the use of each tobacco product, we created models with features consisting of biomarkers of either exposure (for exposure [BoE] classification model) or potential harm (for potential harm [BoPH] classification model). We divided data from current and former smokers into training datasets (80%) and test datasets (20%). These test datasets were evaluated as references for users of electronic cigarettes and smokeless tobacco products. The total number of participants and the number used for machine training are shown in Tables 1 and 2. Using 80% of the training data, the two biomarker classification models were created by machine learning, and used to classify participants into current or former smokers. A random forest model (five-fold, 100-repeated cross-validation) was used for the algorithm. The cross-validation accuracy of the models obtained in the machine learning process was evaluated by the receiver operating characteristic curve-area under curve (ROC-AUC). We input data from the two test datasets on current and former smokers, and also on dual and single users of electronic cigarettes and smokeless tobacco products. The percentage of data classified as current and former smokers was tabulated for each group. There were more current than former smokers and the current smokers were therefore randomly sampled by random number generation, to align the number of participants in each group, because using imbalanced data leads to poorer model performance [22]. The “feature importance” was calculated as the percentage of each feature that influenced the classification into current or former smokers in the cross-validation.

**Table 1:** Demographic information and geometric mean of biomarkers for the exposure biomarkers classification model

			Training data		Test data		Products			
		Full CS^1^	CS^2^	ExSM	test-CS^2^	test-ExSM	dual EPRODS	EPRODS	dual SMKLS	SMKLS
Participants (N)		2189	81	81	21	21	68	142	37	169
Age	Mean	39.1	38.8	30.1	38.7	31.1	36.0	38.1	32.1	42.0
	SD	15.0	14.6	14.8	13.8	14.2	14.2	14.2	15.5	17.0
Gender (N)	Male	1080	35	56	8	12	42	61	-	159
	Female	1109	46	25	13	9	26	81	-	10
Ethnicity (N)	White	1681	65	43	-	-	-	127	-	148
	Black	299	8	27	-	-	-	5	-	6
	Other	209	8	11	-	-	-	10	-	15
Alcohol (N)^3^		1980	76	70	19	19	61	134	32	155
Urban (N)^3^		1957	75	77	18	20	62	134	23	136
High blood pressure (N)^3^		547	23	14	-	-	15	23	9	56
High cholesterol (N)^3^		386	12	15	0	-	9	27	-	41
Diabetes (N)^3^		289	14	6	-	-	6	11	-	23
Cardiovascular disease (N)^4^		230	10	5	-	-	-	10	-	13
Respiratory disease (N)^4^		551	24	11	6	-	10	29	5	28
CEMA (ug/g*CRE)	Mean	368.2	361.0	131.0	278.5	96.0	287.9	169.4	261.1	126.8
	SD	281.0	272.2	97.7	132.3	44.9	186.2	203.7	162.2	93.7
HEMA (ug/g*CRE)	Mean	5.2	5.6	1.7	4.3	1.2	3.9	1.8	3.0	1.2
	SD	6.6	9.9	1.7	3.2	0.8	5.3	1.6	2.6	1.1
HPMA (ug/g*CRE)	Mean	1708.8	1646.3	364.5	1378.5	251.6	1412.2	564.2	1075.3	406.7
	SD	1452.9	1130.8	430.9	1141.4	190.4	1039.8	660.5	740.5	674.5
NNAL (ng/g*CRE)	Mean	410.4	366.8	94.7	355.4	69.7	290.6	76.1	912.5	1480.8
	SD	365.9	247.7	266.6	289.3	159.5	209.8	186.6	1044.4	3142.6
NNNT (ng/g*CRE)	Mean	25.7	24.0	5.6	14.0	3.5	19.2	9.9	40.1	53.1
	SD	78.9	69.7	14.8	12.4	7.6	22.1	16.8	40.3	87.0
P01 (ug/g*CRE)	Mean	86.1	14.5	3.1	12.3	2.1	62.6	44.3	10.3	4.4
	SD	768.7	8.7	4.1	8.0	1.9	375.1	349.9	7.1	20.7
P02 (ug/g*CRE)	Mean	17.2	16.9	7.0	15.5	4.6	15.3	11.5	12.5	8.3
	SD	10.9	7.6	5.7	8.5	2.3	8.1	35.7	5.2	13.4
P10 (ng/g*CRE)	Mean	471.1	386.9	202.0	344.4	354.1	401.8	234.8	399.8	245.4
	SD	1420.5	232.9	154.1	167.6	611.3	410.2	196.2	396.4	236.0
PMA (ng/g*CRE)	Mean	1311.2	1150.4	947.0	1681.7	846.2	1268.6	1543.7	1151.0	1226.2
	SD	1448.9	732.3	817.9	1797.6	510.9	1294.2	1792.3	1028.2	1081.3
TNE7 (ug/g*CRE)	Mean	77.9	73.2	12.1	67.7	6.0	80.2	64.5	98.6	119.4
	SD	51.7	41.8	32.2	37.5	10.6	53.2	53.5	66.8	100.5

*Abbreviations*: *CEMA* N-acetyl-S-(2-carboxyethyl)-L-cysteine, *CS* cigarette smoker, *CRE* creatinine, *dual-EPRODS* user of both conventional and electronic cigarettes, *dual-SMKLS* user of both conventional cigarettes and smokeless tobacco, *ExSM* former smoker, *EPRODS* electronic cigarettes user, *test-CS* test dataset of current smokers, *test-ExSM* test dataset of former smokers, *HEMA* N-acetyl-S-(2-hydroxyethyl)-L-cysteine, *HPMA* N-acetyl-S-(3-hydroxypropyl)-L-cysteinem, *NNAL* 4-(methylnitrosamino)-4-(3-pyridyl)-1-butanol, *NNNT* N’-nitrosonornicotine, *SD* standard deviation, *SMKLS* user of smokeless tobacco products, *TNE7* total nicotine equivalents, *PMA* N-acetyl-S-(phenyl)-L-cysteine, *P01* 1-naphthol or 1-hydroxynaphthalene, *P02* 2-naphthol or 2-hydroxynaphthalene, *P10* 1-hydroxypyrene

^1^Full data of current smokers

^2^Random selection from full data of current smokers to match the number of former smokers for machine learning

^3^Number of participants who answered “Yes” in the questionnaire

^4^Number of participants who answered “Yes” in the relevant disease questionnaire

- In accordance with ICPSR data release rules, tables with cell sizes smaller than the threshold for the specific dataset will not be released

**Table 2:** Demographic information and geometric mean of biomarkers for the potential harm classification model

			Training data		Test data		Products			
		Full CS^1^	CS^2^	ExSM	test-CS^2^	test-ExSM	dual EPRODS	EPRODS	dual SMKLS	SMKLS
Participants (N)		2538	343	343	85	85	82	176	44	198
Age	Mean	39.5	38.8	29.5	37.1	30.0	39.0	39.2	33.4	42.2
	SD	15.0	14.5	13.7	14.0	13.5	15.2	14.9	16.1	16.8
Gender (N)	Male	1217	166	205	40	45	47	74	-	187
	Female	1321	177	138	45	40	35	102	-	11
Ethnicity (N)	White	1975	277	236	62	65	71	154	-	172
	Black	329	40	53	13	12	4	11	-	11
	Other	234	26	54	10	8	7	11	-	15
Alcohol (N)^3^		2280	306	316	78	82	73	165	38	183
Urban (N)^3^		2263	306	336	71	81	74	167	29	164
High blood pressure (N)^3^		659	80	49	20	6	18	28	11	65
High cholesterol (N)^3^		471	50	50	12	6	11	35	5	47
Diabetes (N)^3^		337	50	21	8	-	10	17	-	26
Cardiovascular disease (N)^4^		272	34	17	10	5	9	13	-	16
Respiratory diseases (N)^4^		635	100	61	16	14	18	39	6	28
FIB (mg/dL)	Mean	341.5	347.5	304.6	340.0	307.8	319.0	317.6	317.6	310.4
	SD	103.1	109.8	93.1	101.0	80.6	88.6	102.2	99.8	89.9
hsCRP (mg/L)	Mean	4.0	5.2	2.5	4.2	2.1	3.1	3.2	2.4	3.2
	SD	7.8	10.9	4.2	7.0	3.0	4.1	5.1	3.7	6.6
IL6 (pg/mL)	Mean	2.3	2.5	1.7	2.0	1.4	2.2	1.8	2.5	2.1
	SD	2.1	2.4	1.8	1.9	1.4	2.3	1.8	2.7	2.0
sICAM (ng/mL)	Mean	299.0	307.4	217.3	285.7	218.9	298.6	254.5	272.0	242.0
	SD	113.7	125.8	69.0	119.1	56.5	103.0	90.0	88.6	85.3
8PGFT (ng/g*CRE)	Mean	688.1	682.3	391.1	605.2	417.1	680.6	541.7	560.7	485.9
	SD	403.4	441.2	200.9	232.7	242.4	460.9	348.3	246.7	516.0

Abbreviations: *8PGFT* 8-isoprostane, *CS* cigarette smoker, *CRE* creatinine, *dual-EPRODS* current smoker and user of electronic cigarettes, *dual-SMKLS* current smoker and user of smokeless tobacco, *ExSM* former smoker, *EPRODS* user of electronic cigarettes, *FIB* fibrinogen, *hsCRP* high-sensitivity C-reactive protein, *IL6* interleukin 6, *sICAM* soluble intercellular adhesion module, *SD* standard deviation, *SMKLS* user of smokeless tobacco, *test-CS* test dataset of current smokers, *test-ExSM* test dataset of former smokers

^1^ Full data of all current smokers

^2^ Random selection from data of all current smokers to match the number of former smokers for machine learning

^3^ Number of participants who answered “Yes” in the questionnaire

^4^ Number of participants who answered “Yes” in the relevant disease questionnaire

- In accordance with ICPSR data release rules, tables with cell sizes smaller than the threshold for the specific dataset will not be released

To assess the prevalence of disease in participants classified as current or former smokers, we calculated the percentage of those labelled with CVD or respiratory diseases among those classified as current or former smokers by the classification model, using data from the questionnaire.

Data analysis, including machine learning, was performed in R software version 4.2.1 using the caret package [23].

## Results

### Demographic information and biomarkers

The demographic information and biomarker characteristics of all the participants in this study and those randomly selected for machine learning for the two classification models are shown in Tables 1 and 2. In accordance with ICPSR data release rules, tables with cell sizes smaller than the threshold for the specific dataset will not be released.

### Machine-learning models

**Fig. 1:**
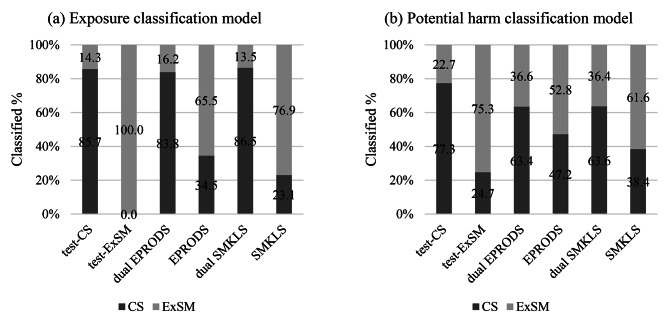
Percentage of users of each tobacco and nicotine product classified as current or former smokers by the two classification models. *Abbreviations*: *CS* current cigarette smoker, *dual-EPRODS* user of conventional cigarettes and electronic cigarettes, *dual-SMKLS* user of conventional cigarettes and smokeless tobacco products, *ExSM* former smoker, *EPRODS* user of electronic cigarettes, *SMKLS* user of smokeless tobacco, *test-CS* models using test data for current smokers, *test-ExSM* models using test data for ex-smokers

**Fig. 2:**
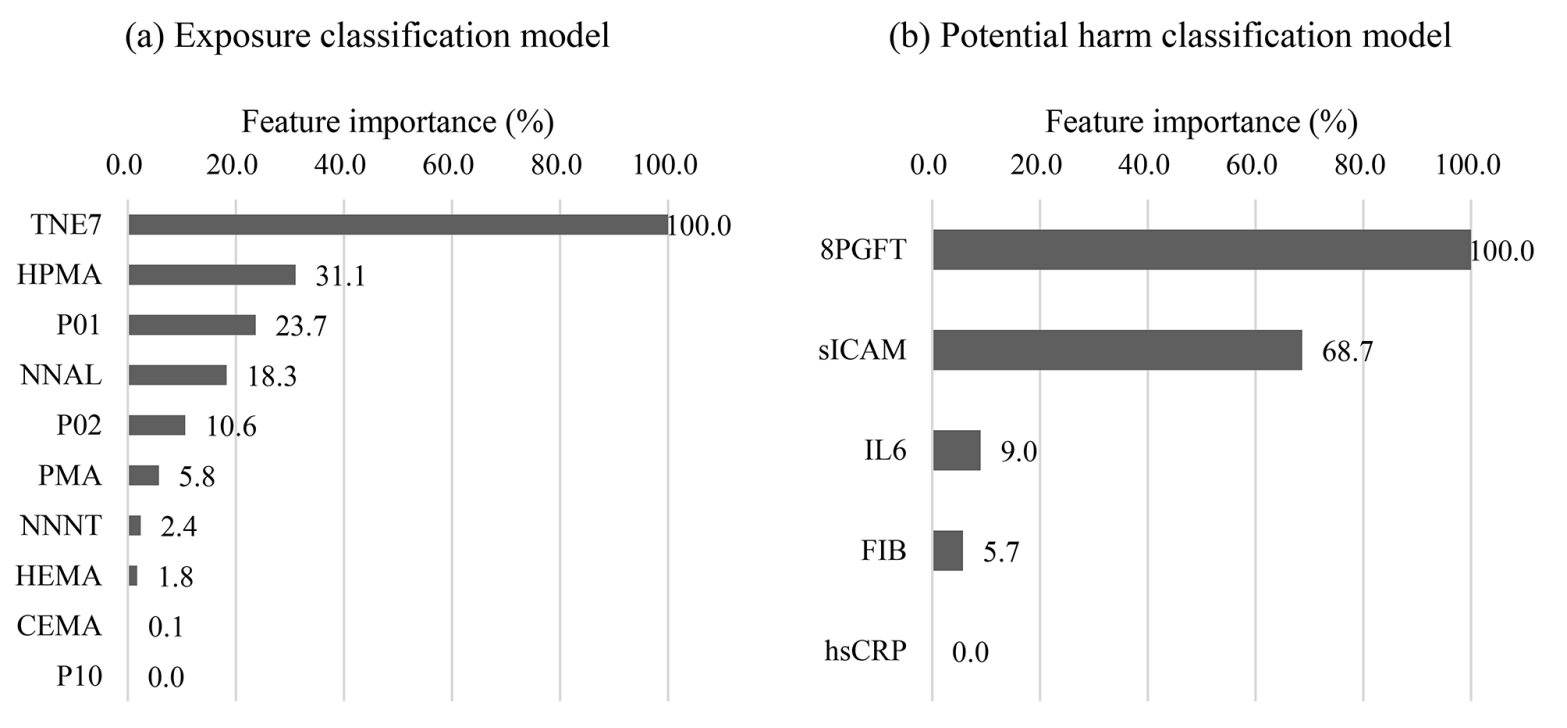
The features contributing to classification as current or former smokers in cross-validation of the exposure and potential harm classification models (feature importance %). *Abbreviations*: *8PGFT* 8-isoprostane, *CEMA* N-acetyl-S-(2-carboxyethyl)-L-cysteine, *FIB* fibrinogen, *HEMA* N-acetyl-S-(2-hydroxyethyl)-L-cysteine, *HPMA* N-acetyl-S-(3-hydroxypropyl)-L-cysteine, *hsCRP* high-sensitivity C-reactive protein, *IL6* interleukin 6, *NNAL* 4-(methylnitrosamino)-4-(3-pyridyl)-1-butanol, *NNNT* N’-nitrosonornicotine, *sICAM* soluble intercellular adhesion module, *TNE7* total nicotine equivalents, *PMA* N-acetyl-S-(phenyl)-L-cysteine, *P01* 1-naphthol or 1-hydroxynaphthalene, *P02* 2-naphthol or 2-hydroxynaphthalene, *P10* 1-hydroxypyrene

The percentage of users of each tobacco product classified as current or former smokers by (a) the biomarkers of exposure model and (b) the biomarkers of potential harm model, and the order of importance of the features are shown in Figs. 1 and 2. The ROC-AUC, a cross-validation model performance score, was 95.0% for the exposure biomarkers classification model and 82.0% for potential harm biomarkers model. For both the classification models, the scores for both test datasets were more than 75%. A higher percentage of users of either electronic cigarettes or smokeless tobacco products were classified as former than current smokers. Both the dual user groups (dual-EPRODS and dual-SMKLS) had a higher percentage classified as current smokers than exclusive users of a single product. In the exposure biomarkers classification model, the most important feature was TNE7, followed by HPMA. In the potential harm biomarkers classification model, 8PGFT and sICAM showed the highest feature importance, followed by IL6.

### Disease prevalence

Among those indicated by either classification model as a current or former smoker, we calculated the percentage with CVD or respiratory diseases for each group. The percentage with CVD or respiratory diseases was consistently higher among those classified as current than former smokers in both models (Table 3).

**Table 3:** The percentage of participants classified as current or former smokers by exposure or potential harm classification models who had cardiovascular or respiratory diseases

	Predicted by exposure classification model		Predicted by potential harm classification model	
	Current smoker	Former smoker	Current smoker	Former smoker
Cardiovascular disease	9.9%	6.4%	10.9%	6.3%
Respiratory diseases	19.4%	16.7%	22.2%	14.2%

## Discussion

We first developed the biomarkers of exposure-based classification model to classify participants as either current or former smokers. The result should reflect the overall exposure level to constituents of cigarette smoke. This model predicted that users of electronic cigarettes and smokeless tobacco products would be more like former than current smokers, suggesting that the use of these products reduced exposure. Consistent with previous reports [18, 20], TNE7, the total amount of nicotine metabolites [24], was identified as the most important variable in distinguishing between current and former smokers. TNE7 was much lower in former than current smokers. However, the level in users of either electronic cigarettes or smokeless tobacco products was higher than in current smokers, suggesting that the classification of these two groups did not depend on the TNE7 level but on other biomarkers of exposure. High TNE7 levels in users of potentially lower-risk tobacco products are expected because they provide access to the parent chemical (i.e., nicotine). It is therefore likely that other biomarkers of exposure contribute more to the classification of users of electronic cigarettes and smokeless tobacco products.

Similarly, the classification model based on biomarkers of potential harm also classified users of electronic cigarettes and smokeless tobacco products as more like former than current smokers, but to a lesser extent than the exposure model. The difference in the results between users of electronic cigarettes and smokeless products from the two different classification models may be due to the difference in characteristics as biomarkers. The exposure biomarkers are for cigarette smoke-specific constituents, but the biomarkers of potential harm could be influenced by a variety of factors other than smoking. The most important features of the potential harm model were 8PGFT and sICAM. Both biomarkers are known to be associated with oxidative stress and/or inflammation [25, 26] and also recognized as predisposing factors for CVD [27, 28]. IL-6 was also important in the potential harm biomarkers model. IL-6 has a short half-life and inter-individual variability, and its association with risk for CVD and respiratory diseases is still controversial. However, it is among the most frequently studied biomarkers and several studies have identified its importance for disease risk [29, 30]. The biomarkers of potential harm are important, but no single one can explain all disease risk. Our model therefore includes several biomarkers of potential harm, and could approximate the relative disease risk between cigarette smoking and smoking cessation. Our potential harm classification model is based on the hypothesis that smoking cessation could minimize the risk of smoking-associated diseases. If a user of potentially lower-risk tobacco products is classified as a former smoker based on their biomarkers of potential harm, this suggests that using these products does indeed reduce the disease risk compared with smoking conventional cigarettes. Our results therefore suggest that both electronic cigarettes and smokeless tobacco products could contribute to reduce smoking-associated disease risk. Disease prevalence was slightly lower in participants classified as former smokers than those classified as current smokers. Our results show that participants classified as current smokers in either model had relatively higher rates of CVD or respiratory diseases than those classified as former smokers. There may therefore be a reduced risk of disease among users of potentially lower-risk products. The results of the feature importance, and of previous reports of biomarkers, suggest an association with CVD.

We also evaluated dual users of both conventional cigarettes and either electronic cigarettes or smokeless tobacco products. The exposure model recognized these dual users as current smokers. If participants use both cigarettes and electronic cigarettes or smokeless tobacco products on a daily basis, the biomarkers of exposure could be closer to those of cigarette smokers than those who use only potentially lower-risk products. The result of the exposure model reflects this hypothesis. However, the model based on biomarkers of potential harm showed an intermediate result for dual users, between smokers and users of only electronic cigarettes or smokeless tobacco products, suggesting that dual use reduces the risk of harm. However, longitudinal observation is necessary to detect significant changes in biomarkers of potential harm, and to establish whether robust homeostasis in the human body suppresses rapid changes. Multiple exposures and cumulative effects possibly contribute to the changes in the biomarkers of potential harm as well as the manifestation of health impacts. The extent of risk reduction in dual users would therefore depend on the proportion of cigarette smoking and use of electronic cigarettes and smokeless tobacco products.

This study had several limitations. In this study, we used strict definitions for each group of tobacco product users. Exclusive users had to use only one product on a daily basis. Users of electronic cigarettes and smokeless tobacco products who also smoked were defined as dual-users. Some similarities were seen between users of electronic cigarettes and smokeless tobacco products and former smokers in the population used for modeling. However, this should be further investigated with more study participants to reflect the real-world population. The health impact of e-cigarettes is still controversial [31, 32] and the subject of ongoing study, and we cannot conclude that the risk of disease is reduced even if e-cigarette users were more likely to be classified as former smokers in this study. The variables used in this study are absent from the succeeding waves, and we therefore cannot evaluate the time-related changes in the classification results. However, longitudinal analyses could provide more insights into the health impact of these new tobacco products because the pathogenesis of smoking-related chronic diseases takes time to manifest. Expansion of variables to include items such as “Frequency of product use” and “Start of product use” could also provide additional insights, although we could not obtain sufficient data for this analysis from Wave 1 alone. These limitations could be addressed if the variables used in this study are available in future waves of the PATH study.

## Conclusion

The results of our classification models based on biomarkers of exposure and potential harm showed that the biomarker profiles of people who used electronic cigarettes or smokeless tobacco products were more like those of former than current smokers. This suggests that exposure to constituents in cigarette smoke and the resulting biological effects have potential to be reduced with the use of these products, at least among those involved in the PATH study.

## Data Availability

Data are available in a public, open-access repository, the National Addiction and HIV Data Archive: 10.3886/Series606.

## Abbreviations

***8PGFT***: 8-isoprostane
***BoE***: biomarkers of exposure
***BoPH***: biomarkers of potential harm
***CEMA***: N-acetyl-S-(2-carboxyethyl)-L-cysteine
***COPD***: chronic obstructive pulmonary disease
***CS***: cigarette smokers
***CVD***: cardiovascular disease
***EPRODS***: electronic cigarettes
***ExSM***: former smokers
***FDA***: Food and Drug Administration
***FIB***: fibrinogen
***HEMA***: N-acetyl-S-(2-hydroxyethyl)-L-cysteine
***HPHC***: harmful and potentially harmful constituent
***HPMA***: N-acetyl-S-(3-hydroxypropyl)-L-cysteine
***hsCRP***: high-sensitivity C-reactive protein
***ICPSR***: Inter-University Consortium for Political and Social Research
***IL6***: interleukin 6
***NIH***: National Institutes of Health
***NNAL***: 4-(methylnitrosamino)-4-(3-pyridyl)-1-butanol
***NNNT***: N’-nitrosonornicotine
***PATH***: Population Assessment of Tobacco and Health [study]
***PMA***: N-acetyl-S-(phenyl)-L-cysteine
***P01***: 1-naphthol or 1-hydroxynaphthalene
***P02***: 2-naphthol or 2-hydroxynaphthalene
***P10***: 1-hydroxypyrene
***RESP***: respiratory diseases
***sICAM***: soluble intercellular adhesion module
***SMKLS***: smokeless tobacco products
***TNE7***: total nicotine equivalents
***ROC-AUC***: receiver operating characteristic curve-area under curve
